# Assessing the Effect of Nonvisual Information Factors in Pandemic-Related Video Communication: Randomized Controlled Between-Subjects Experiment

**DOI:** 10.2196/42528

**Published:** 2023-08-23

**Authors:** Daniel Adrian Lungu, Jo Røislien, Siv Hilde Berg, Ionica Smeets, Marie Therese Shortt, Henriette Thune, Kolbjørn Kallesten Brønnick

**Affiliations:** 1 SHARE – Centre for Resilience in Healthcare, Department of Quality and Health Technology Faculty of Health Sciences University of Stavanger Stavanger Norway; 2 Science Communication and Society, Institute of Biology Leiden University Leiden Netherlands

**Keywords:** video communication, COVID-19, trust, comprehension, intentions, behavior, visual, pandemic, risk, communication, policy, behavior, effect, video, experiment

## Abstract

**Background:**

Videos have been an important medium for providing health and risk communication to the public during the COVID-19 pandemic. Public health officials, health care professionals, and policy makers have used videos to communicate pandemic-related content to large parts of the population. Evidence regarding the outcomes of such communication, along with their determinants, is however limited.

**Objective:**

The aim of this study was to test the impact of nonvisual information factors of video communication on 4 outcomes: trust, comprehension, intentions, and behavior.

**Methods:**

Twelve short health communication videos related to pandemics were produced and shown to a large sample of participants, applying a randomized controlled between-subjects design. Three factors were included in the creation of the videos: the topic (exponential growth, handwashing, and burden of pandemics on the health care system), the source (expert and nonexpert), and a call to action (present or absent). Participants were randomly assigned to 1 video intervention, and 1194 valid replies were collected. The data were analyzed using factorial ANOVA.

**Results:**

The 3 pandemic-related topics did not affect trust, comprehension, intentions, or behavior. Trust was positively influenced by an expert source (2.5%), whereas a nonexpert source instead had a positive effect on the proxy for behavior (5.7%) compared with the expert source. The inclusion of a call to action had a positive effect on both trust (4.1%) and comprehension (15%).

**Conclusions:**

Trust and comprehension in pandemic-related video communication can be enhanced by using expert sources and by including a call to action, irrespective of the topic being communicated. Intentions and behavior appear to be affected to a small extent by the 3 factors tested in this study.

**International Registered Report Identifier (IRRID):**

RR2-10.2196/34275

## Introduction

### Background

Videos have been an important medium for providing health and risk communication to the public during the COVID-19 pandemic [1,2]. Public health officials, health care professionals, and policy makers have used videos to communicate pandemic-related content to large parts of the population. However, pandemic health communication videos made by health authorities have been found to lack in creativity and have limited reach [3]. Although research has identified certain traits of successful health communication, sparse research exists on the association between video features and the *outcomes* of such communication [4].

The modified Integrated-Change Model [4,5] highlights how the 4 dimensions of health communication outcome (emotion, awareness, motivation, and action) are influenced by both factors related to the communication (eg, design of message and choice of source) and factors inherent in the receiver (eg, biological and psychological factors). Although the latter factors are important when creating effective and targeted communication, they cannot be manipulated by the communication creators. The information factors, however, can. How effectful it is to tweak these factors is largely unknown.

This study addresses the research gap regarding the effect of nonvisual information factors on communication outcomes. The aim of the study is to assess the effect of 3 aspects of the messenger (source) and the message (topic and call to action) on the outcomes (trust, comprehension, intentions, and behavior) of pandemic video communication. Therefore, the source, topic, and call to action are the independent variables, whereas trust, comprehension, intentions, and behavior are the dependent variables of our study. The rationale for the choice of variables is described in the following paragraphs.

### Messenger and Source Factors

*The source* refers to the origin of the information being communicated. This can be an expert source (eg, research organizations, universities, and government departments) or a nonexpert one (eg, colleagues, friends, and word of mouth). Although expert sources are constituted in some regularized or legal manner in relation to the user, nonexpert sources have no such basis [6]. The choice of presenter used in a video is found to be an important factor in gaining viewers’ trust [7]. Trust in scientists and health experts is generally high [8,9], and health authorities often use field experts as presenters [3]. Nevertheless, scientists and field experts are frequently used as presenters in public communication, despite generally being untrained in mass media communication [10].

### Message Factors

Although the messenger is important, tailored message *topics* are considered the core of health communication. It is through messages we construct, modify, and maintain meanings of health with the audience [11-13]. Messages influence both individuals and groups, and through societal influence, messages are capable of changing norms and policies [14,15]. To succeed with health communication, tailored messages are needed [12,13,16-18].

During the response phase of the recent COVID-19 pandemic, the importance of washing hands, understanding rapid spread and exponential growth, and the potentially negative impact of several infected people on the capacity of health care systems were among the topics communicated to the population.

The importance of washing hands was highlighted by the World Health Organization both through developing guidance [19] and in press conferences during the early stage of the pandemic [20,21]. Multiple research articles later contributed to reiterating the role of handwashing in infection prevention [22,23].

The potential for extreme growth in cases of a pandemic is linked to the mathematical phenomenon of exponential growth. Exponential growth is fiercely difficult to communicate [24]. Yet, exponential growth and the accompanying basic reproduction number have been among the most common metrics used by policy makers and scientists to communicate whether the spread of COVID-19 is increasing or decreasing in magnitude. The reproduction number has been used frequently by policy makers and scientists to communicate about the COVID-19 pandemic to the public and to make decisions regarding issues such as mandatory social distancing, use of face masks, and lockdowns [25].

The number of hospitalized patients with COVID-19 challenged the capacity of hospitals to admit patients and deliver care. This was the case worldwide, for acute and intensive care beds, both for patients with COVID-19 and those without COVID-19. Standard health care operations were put at risk, and in some cases, elective and planned surgical activity was postponed because of this lack of capacity [26-29]. Capacity constraints were at the core of the discussion also in Norway, and policy makers tried to openly communicate this to the public, with the aim of providing explanations for the reasoning behind restrictive measures [30].

Health communication strives to support and empower rather than convince the public [31]. Providing people with fact-based information, which helps them make informed choices, is considered the gold standard [32,33]. Authorities may however seek to recommend people to follow recommendations in a crisis situation, not merely informing the public [34]. Yet, being too persuasive could reduce compliance and public trust [34]. *A* call to action has been suggested as a further means for changing people’s behavior [35,36]. Calls to action are used in marketing to tell or encourage prospects and website or social media users what to do with the information they have been presented with. To be effective, calls to action should be valuable, easy to use, prominent, and action oriented [37].

### Outcomes

Generally, we want our health communication to affect the receiver in one way or another, be it to understand something (eg, exponential growth) or to do something (eg, to wash hands).

Trust has been shown to influence self-reported intention to act upon health advice [38-40]. People tend to have trust in health care professionals as spokespersons and information sources in public health emergencies [40], and research on pandemics has shown that trust in formal sources has been associated with more accurate pandemic risk knowledge and self-protective behavior [41].

Comprehension refers to the ability to understand the information and to incorporate it into one’s knowledge. Several barriers and facilitators to comprehension have been identified. They can be classified into 3 categories: patient-specific, physician-specific, and other factors [42,43]. Health literacy is the most frequently indicated determinant of comprehension of health information [44-47], whereas other determinants include printed-versus-digital information [48], message complexity [49], and use of illustrations [50].

Intentions go beyond mere knowing [51-55]. In order to define our behavioral outcome measures, we relied on the Theory of Planned Behavior that links beliefs to behavior [51]. More specifically, it states that the 3 main components of beliefs—attitude, subjective norms, and perceived behavioral control—define intentions. Consequently, the theory affirms that intentions are the most adjacent proxy of human social behavior. Intentions have been a main outcome investigated during the recent COVID-19 pandemic: intentions to vaccinate [56-58], to self-isolate [59], to keep the social distance, and to follow the “stay at home” policy [60,61].

Although intentions may be a proxy of behavior, the limitations of intentions as a predictor of behavior have long been debated by international literature [62,63]. Although the public’s intentions are highly valuable for public health practitioners and policy makers, Sheeran and Webb [64] analyzed the intention-behavior gap and concluded that realization from intentions into action occurs in approximately 50% of the time. This rate appears to be influenced by the quality of the intention, the nature of the goal, and the basis and properties of intention.

## Methods

### Design

This study applies Berlo’s communication process model to behavioral theories [65]. The model defines communication as a process involving 4 key elements: a source, a message, a channel, and a receiver. On the basis of these elements, we conducted group discussions within the COVCOM (Creating Effective, Evidence-Based Video Communication of Public Health Science) research project team regarding the most salient factors to pandemic communication [66], and we decided to explore 3 key features of pandemic video communication: messenger or source, message, and message tone. Although the channel (video) and the receiver (study participants from the public) were defined, the source and the content and tone of the message were manipulated and tested.

The messenger or source had 2 categories: expert and nonexpert. Although the same actor was used for all videos, the messenger or source was manipulated by using text over image on the bottom left of the screen, introducing the presenter with name and profession. In the expert case, he was introduced as an infectious disease field expert, whereas in the nonexpert one, he was represented as a salesman.

The topic had 3 categories: exponential growth, handwashing, and the impact of pandemics on the health care system. The selection of topics was made based on the results of an interview study of expert opinions performed by our research group, and that identified exponential growth, handwashing, and the effect of a pandemic on the health care system as key topics to be communicated [31]. Although other topics could have been relevant to study as well, time and resources constraints led us to choose the 3 most relevant ones.

The message tone differed by the inclusion, or not, of a call to action at the end, thus having 2 categories: present (Y) or absent (N). In the version without a call to action, the video merely explained the topic, whereas in the version containing a call to action, a final motivational message calling to an individual and collective action to defeat pandemics was included at the end.

The 3 variables were modeled as factors in a full factorial between-subjects randomized controlled experiment. Communication is not a series of independent factors but rather the combination of multiple interwoven factors, and a factorial design was thus adopted to incorporate the possibility of interaction between the 3 features under study.

Therefore, the experiment used a 2 × 3 × 2 full factorial design. An overview is presented in Table 1.

**Table 1:** Overview of the factorial design of the experiment.

Topic^a^	Call to action, n			
	1. Present		2. Absent	
	1. Expert	2. Nonexpert	1. Expert	2. Nonexpert
1. Exponential growth	111	112	121	122
2. Handwashing	211	212	221	222
3. Impact of pandemics on the health care system	311	312	321	322

^a^The 3-digit code denotes all 12 individual combinations of the 3 experimental factors.

### Video Creation

A professional scriptwriter was hired to create 12 scripts covering both the 3 different topics and the variations dictated by the factorial design. After 2 rounds of revision, the scripts were approved by both the researchers and the writer.

**Figure 1:**
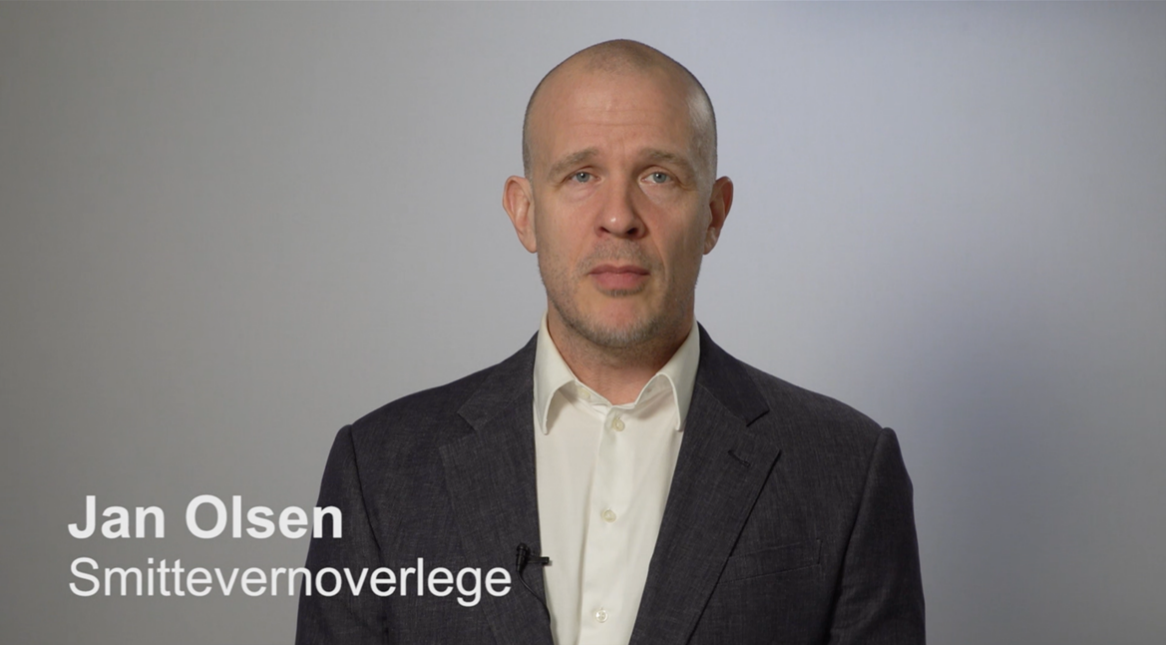
Thumbnail of one of the videos (expert source—infectious diseases specialist).

Based on the scripts, 12 videos were produced in collaboration with the department for development of digital learning resources of the University of Stavanger. To avoid confounding factors, all other variables besides the experimental ones were controlled for: the same professional actor was used to shoot all videos, wearing the same clothing (white shirt and dark gray suit), placed in front of a neutral gray background (Figure 1). All videos were of approximately the same length (range 00:54-01:25). Videos are stored in the OpenScience repository of the University of Stavanger [67].

### Participants

Participants were recruited among the members of the Norwegian Air Ambulance Foundation (NAAF), one of the partners of the COVCOM project [66]. An a priori power analysis assuming a 1-way ANOVA with 12 groups, a medium effect size (*f*) of 0.25, and 95% statistical power revealed that the minimum sample size was 35 participants per arm, 420 in total. From previous experiences, response rates of the NAAF members were lower than 10%, even with reminders. Therefore, we adopted a conservative approach and invited 12,000 people to participate, so that a response rate of 3.5% would be sufficient to reach the minimum sample size. The 12,000 people who were invited were randomly assigned to 1 of the 12 video interventions, with the criteria of having balanced groups in terms of age distribution, female-male proportion, and geographical distribution. Members received an invitation email with a short description and a link to an external page to participate.

Among the invited participants, 54% (6480/12,000) were male and age ranged from 18 to 90 (median 64.8, IQR 58-73) years.

### Data Collection and Management

The data collection phase was managed by the NAAF’s IT infrastructure. Their familiarity of members with the sender’s email address would reduce the risk of emails being misdirected to the spam folder and complied with the General Data Protection Regulation and the national regulations of not sharing information about members with another organization. The data collection lasted from June 1, 2021, through June 9, 2021.

Emails sent to the NAAF members contained 1 of the 12 videos, and a link leading to 1 of the 12 surveys created on the SurveyMonkey platform. The data collection was anonymous by enabling this option in SurveyMonkey, and therefore the IP addresses of respondents were not collected. The email addresses were used only for the invitation and were not linked to the collected responses. No personal or sensitive information was asked of respondents.

Each response has been attributed a unique identifier, and collected responses were stored in a separate database unlinked from the NAAF members’ database. In order to leave open the opportunity to conduct a longitudinal study with a follow-up after 12 months, respondents were asked to digit their email address in case they wanted to participate further.

### Ethical Considerations

The legal department of the NAAF confirmed that the study is compliant with the General Data Protection Regulation and national regulations, and as no personal or sensitive data were collected or processed, approval from the Norwegian Centre for Research Data or from the Ethical Committee was not necessary.

### Measures

The questionnaire contained questions on the following outcome measures: comprehension, trust, past and future intentions, and a proxy for behavior. Comprehension was measured by 1 close-ended question regarding the content of the video (4 alternatives, of which 1 was correct). Trust was measured through a 2-item 5-point Likert scale developed by Sillence et al [40]. The scale intends to measure trust in web-based health information and advice, and it is suitable for the aim of the study as participants are receiving web-based health information and advice. Intentions were measured by one 6-point Likert scale item. The proxy for behavior consisted of a question asking participants to fill in their email addresses in order to be further part of a pandemic research project. The questionnaire also included sociodemographic characteristics of respondents: age, gender, education level, income level, and whether they live in a big city, a town, or rurally. The questionnaire further contained attention checks and the Belief in Science Scale [68] to define a baseline of the level of scientific belief of respondents. The Belief in Science Scale is a measurement tool of attitudes toward science, where science shares similarities with religion in terms of the comforting role it plays in individuals’ lives.

The complete questionnaire (both in English and Norwegian) is available in Multimedia Appendix 1.

### Data Analysis

The data analysis was conducted using standard factorial design statistics to analyze main effects and interaction effects using between-groups variance analysis (factorial ANOVA). Main effects and all interactions between the 3 video factors were investigated. Pairwise comparisons were performed, and the Bonferroni correction was used to control for the family-wise error rate. The analysis was performed in RStudio (Posit). *P* values below .05 were considered statistically significant.

## Results

From the 12,000 emails sent, we collected 1194 complete replies, corresponding to a response rate of 9.97%. After removing 2 responses that failed the attention checks, a total of 1192 valid responses were included for analysis. As the aim of the study was to compare video factors by assigning participants randomly to 1 of the 12 video versions, and randomization was performed after inclusion, the relatively low response rate is not a limitation.

For the full factorial design, with its 12 combinatorial possibilities, the number of respondents in each factorial combination ranged from 93 (7.80%) to 109 (9.14%) of the 1192 valid responses.

Regarding the topic, 412 (34.56%) respondents watched a video about exponential growth, 383 (32.13%) watched a video about handwashing, and 397 (33.31%) watched a video about the burden of the pandemic on the health care system. A total of 605 (50.75%) of respondents watched a video with an expert messenger or source, and 587 (49.25%) watched a video with a nonexpert one. A total of 621 (52.10%) respondents watched a video with a call to action, and 571 (47.90%) watched a video without one. An overview of outcome measures by video version is presented in Table 2.

**Table 2:** Outcome results by video version.

Version	Trust 1-6, mean (SD)	Comprehension 0-1, mean (SD)	Intentions 1-6, mean (SD)	Behavior 0-1, mean (SD)
111	5.26 (0.59)	0.78 (0.42)	5.44 (0.59)	0.32 (0.47)
112	5.38 (0.63)	0.77 (0.42)	5.50 (0.59)	0.20 (0.40)
121	5.44 (0.57)	0.72 (0.45)	5.65 (0.48)	0.32 (0.47)
122	5.50 (0.65)	0.78 (0.42)	5.54 (0.63)	0.29 (0.45)
211	5.09 (0.90)	0.81 (0.40)	5.61 (0.57)	0.35 (0.48)
212	5.29 (0.54)	0.81 (0.40)	5.64 (0.62)	0.28 (0.45)
221	5.33 (0.64)	0.80 (0.40)	5.56 (0.52)	0.37 (0.49)
222	5.53 (0.50)	0.99 (0.10)	5.52 (0.50)	0.32 (0.47)
311	5.51(0.60)	0.82 (0.39)	5.67 (0.49)	0.28 (0.45)
312	5.20 (0.66)	0.47 (0.50)	5.51 (0.59)	0.34 (0.48)
321	5.35 (0.78)	0.56 (0.50)	5.61 (0.49)	0.27 (0.45)
322	5.49 (0.62)	0.98 (0.14)	5.48 (0.56)	0.27 (0.45)
Total	5.37 (0.65)	0.78 (0.41)	5.56 (0.56)	0.30 (0.46)

Trust was generally high, with a mean value of 5.37 on a 1-6–point scale. There was little variation between the groups, with a range of 5.09 to 5.53. Comprehension was measured as the percentage of correct answers and therefore ranges between 0 and 1, where 1 is equal to 100%. The mean value of the sample was 0.78, with significant between-groups variation: only 47% of respondents who watched video version 312 understood the information, whereas for version 222, almost all respondents (99%) comprehended it. Intentions to follow pandemic recommendations were high (mean value of 5.56 on a 1-6 scale), with little between-groups variation (range 5.44-5.67). The measured proxy for behavior was generally low (mean 0.30), with some between-groups variation (range 0.20-0.37).

An overview of the results of the factorial ANOVA is presented in Table 3.

**Table 3:** Results from factorial ANOVA.

Variable	Outcomes							
	Comprehension		Trust		Intentions		Behavior	
	Effect, estimate (CI) or *F* test (*df*)^a^	*P* value	Effect estimate (CI) or *F* test (*df*)	*P* value	Effect estimate (CI) or *F* test (*df*)	*P* value	Effect estimate (CI) or *F* test (*df*)	*P* value
Topic	0	.09	0	.09	0	.07	0	.48
Action	0.150 (0.105-0.195)	*<.001* ^b^	0.207 (0.133-0.280)	*<.001*	0	.56	0	.55
Source	0	.24	0.127 (0.053-0.201)	*.001*	0	.22	0.057 (0.005-0.110)	*.03*
Topic × Action	48.115 (2)^a^	*<.001*	0^a^	.59	0^a^	.053	0^a^	.71
Topic × Source	0^a^	.74	0^a^	.47	0^a^	.38	0^a^	.47
Action × Source	0^a^	.96	0^a^	.38	0^a^	.44	0^a^	.25
Topic × Action × Source	0^a^	.47	0^a^	.87	0^a^	.27	0^a^	.57

^a^*F* test (*df*) values used.

^b^Italicized *P* values represent significance *P*<.05.

The model yielded statistically significant results for the main effect of call to action (*P*<.001) and the interaction effect between call to action and topic (*P*<.001) on comprehension. The pairwise comparison showed a statistically significant (*P*<.001) effect difference of call to action on comprehension. The difference was 0.15, meaning that including a call to action led to an increase of 15% on comprehension.

The analysis showed a statistically significant main effect of call to action (*P*<.001) and source (*P*=.001) on trust, whereas none of the interaction effects were statistically significant. The pairwise comparison showed a statistically significant (*P*<.001) effect difference of call to action on trust. The difference was 0.207 on a scale of 5, meaning that including a call to action led to an increase of 4.14% on trust. The pairwise comparison showed a statistically significant (*P*=.001) effect of source on trust. The difference was 0.127, meaning that an expert source led to an increase of 2.54% on trust compared with a nonexpert one.

The analysis did not show any statistically significant main effect of the topic on one of the outcomes, and no significant interaction effect was revealed either.

The analysis showed a statistically significant main effect of the source (*P*=.03) on behavior, whereas none of the interaction effects were statistically significant. The pairwise comparison showed a statistically significant (*P*=.03) effect of 0.057, meaning that an informal source led to an increase of 5.7% on behavior with respect to a formal source. The effect of the duration of the video (minimum 0:54, maximum 1:25) was not statistically significant (*P*>.3) for all outcomes.

## Discussion

### Principal Findings

Effective mass communication is key during a critical event like a worldwide pandemic. When used right, video is an effective medium for reaching out to large portions of the public. While the importance of visual and creative means in reach was demonstrated for COVID-19 pandemic–related videos [3], there was less evidence regarding nonvisual elements. This study demonstrates the effects of various nonvisual information factors on 4 different outcomes of pandemic video communication: trust, comprehension, intention, and behavior. Some of the findings support existing evidence, whereas others provide novel insights for the understanding of the effectiveness of pandemic video communication.

### Principal Results

Trust is key in any communication [69-71], thus also during a pandemic where believing that the government acts in your best interest or not is at stake [72]. The topic being communicated did not have any effect on trust. However, whether the message comes from an expert or nonexpert did have an effect on trust. The positive effect of a formal expert source on trust is aligned with international literature [7,73]. Although relatively small (2.54%), the effect is yet relevant as the source was manipulated only by the means of a simple text over in the video introducing the presenter. All other variables were kept unchanged, for example, the presenter, attire, background, setting, use of props, and style of language. Manipulating all these factors could in sum contribute to a considerable increase in trust.

The inclusion of a call to action at the end of the message also increased trust. The inclusion of a call to action may impact the level of cognitive processing by leading to elaborations, which might increase the feeling of knowing and hence trust. Notably, the use of a call to action is not unproblematic, seen from a health communication perspective. Multiple papers comment on the importance of being neutral when handing over information so that recipients can make informed choices—“strive to inform, never persuade” [74]. According to Oxman et al [34], persuasion should be seen as a continuum from information to coercion. A call to action involves recommending people how to behave based on explicit reasons, which is at the noncoercive end of the continuum. Notably, the call to action increased trust, and our results thus indicate that health science communication might afford to be somewhat more instructional than previously believed [75,76].

Comprehension, on the other hand, was not significantly affected by whether the source was a formal field expert or a nonexpert. This is in line with previous works regarding the source as a potential determinant of comprehension [77-79]. Further, we did not find any effect of the topic on comprehension. The comprehension score was relatively high (78% correctly understood the message conveyed) with little differences between the 3 topics, indicating that people find the topics equally and relatively easy to understand. The inclusion of a call to action, however, led to an increase in comprehension by 15%. This can be explained by the role that calls to action have in motivation and in turn by the relationship between motivation and comprehension [80]. Therefore, the call to action may have triggered listeners’ motivation and increased their comprehension of pandemic health communication.

As with comprehension, intention to follow the pandemic recommendations was not influenced by whether the source was a field expert or a nonexpert. More surprisingly, however, although the inclusion of a call to action increased comprehension, it did not increase intention. Although the factorial ANOVA showed a main effect of the topic of intentions to follow recommendations, the pairwise comparisons did not yield any statistically significant difference between the 3 topics. That is, none of the investigated variables were found to have an effect on intention.

As the intended outcome of health communication during a public health crisis like a pandemic is often not merely informing the public but also wanting them to act in a certain way in order to reduce risk for themselves and society, some of our findings might come across as depressing. Greater intention to follow pandemic recommendations was expected if the communication comes from an infectious diseases specialist with respect to a sales consultant. Furthermore, also the inclusion of a call to action was expected to lead to greater intention to follow recommendations. Both these factors increase trust in our experiment, but, alas, not intention. On the other hand, we observed little variation in the data, along with a ceiling effect. The mean value for intention to follow the recommendations was 5.56 on a 1-6 scale, with a group mean ranging from 5.44 to 5.67 (high to low ratio equal to 1.04). The small variation in the data might hide the investigated effects. Further research in different contexts is encouraged.

As with intention, neither the topic nor the inclusion of a call to action had a significant impact on behavior. As behavior is a “harder” outcome than mere intentions, this comes as no surprise. The source, however, had a statistically significant impact on behavior, with the nonexpert source leading to an increase of 5.7% in the number of people who decided to fill in their email addresses in order to continue being part of the pandemic research project. Although this can be explained by a higher identification of participants with a nonexpert source compared with a field expert, this finding requires careful interpretation. First, as opposed to the high mean for intention, the mean for behavior was relatively low (0.30 on a 0-1–point scale), with a group mean ranging from 0.20 to 0.37. Second, the proxy used to measure behavior—asking participants to fill in their email address to receive updates and advice—might be inaccurate in measuring the desired construct. As most indirect measures, caution is required in drawing conclusions. The literature is scarce in this point. A recent literature review revealed that only a handful of studies assessing the association between health communication and actual behavior have been carried out [4]. More research in this field is thus needed.

### Limitations

Our study comes with a few limitations in addition to the proxy for behavior addressed in the discussion. The scales used to measure subjective comprehension and intentions are made of a limited number of items and therefore might not be specific enough to be able to measure the desired construct. Moreover, the research was performed in Norwegian and with Norwegian participants. Norway is a high-trust society, and therefore findings might be context-specific and not generalizable to other countries. Moreover, participants are members of the NAAF—a nonprofit organization delivering advanced lifesaving medical treatment and supported by more than 300,000 members. As the sample used in this study comprised the NAAF members, it cannot be considered a random sample and therefore might not be representative of the general population in terms of education and age distribution. These limitations, along with the little variation and ceiling effects observed for some variables, open a research opportunity for scholars worldwide.

### Conclusions

The source, topic, and call to action showed mixed effects on the outcomes of pandemic video communication. Comprehension and trust were positively impacted by using an expert source (ie, an infectious disease field expert), as opposed to a nonexpert source (ie, a sales consultant), and by including a call to action, irrespective of the topic. Adding a recommendation for how to behave in addition to the explicit reasons for doing so does not erode people’s trust. These findings are relevant for public health communicators and policy makers who need citizens to comprehend and trust messages conveyed to them. At the same time, the same nonvisual information factors (source, topic, and call to action) had limited or no effect on the intention to follow pandemic recommendations and behavior for the participants to this study. Further research should focus on what determines people to follow public health recommendations and behave accordingly in a time of a crisis.

## Appendix

Multimedia Appendix 1 Questionnaire in English and Norwegian.

Multimedia Appendix 2 CONSORT checklist.

## Abbreviations

COVCOM: Creating Effective, Evidence-Based Video Communication of Public Health Science
NAAF: Norwegian Air Ambulance Foundation

## Data Availability

All videos are stored in the OpenScience repository of the University of Stavanger [67].
