# Supplementary material for: Assessing the Effect of Nonvisual Information Factors in Pandemic-Related Video Communication: Randomized Controlled Between-Subjects Experiment
Source: J Med Internet Res. 2023 Aug 23;25:e42528. doi: 10.2196/42528 (PMC10483294; doi:10.2196/42528)
Supplement: Multimedia Appendix 1 [file jmir_v25i1e42528_app1.docx]

Multimedia Appendix 1

Questionnaire (In Norwegian)

*Kjære deltaker, takk for ditt bidrag til denne forskningen. Vi setter pris på at du tar ti minutter av tiden din til å hjelpe oss. Se den korte videoen nedenfor. Deretter følger noen få korte spørsmål. Svarene dine er anonyme og vil bli analysert i samlet form. Du vil bli oppdatert om funnene så snart resultatene vil være tilgjengelige.*

1. Fokuset på videoen du har sett var:

a. artistisk

b. vitenskapelig

2. Taleren i videoen var:

a. ei dame

b. en mann

| **Utsagn** | Svært uenig | Uenig | Litt uenig | Litt enig | Enig | Svært enig |
| --- | --- | --- | --- | --- | --- | --- |
| 3. Jeg skjønte innholdet i videoen | 1 | 2 | 3 | 4 | 5 | 6 |
| 4. Budskapet var klart og tydelig | 1 | 2 | 3 | 4 | 5 | 6 |
| 5. Jeg husker informasjonen | 1 | 2 | 3 | 4 | 5 | 6 |
| 6. Jeg kan viderefortelle budskapet | 1 | 2 | 3 | 4 | 5 | 6 |

7. Hvis R=0.7, hvor mange blir smittet av 100 positive?

a. 7

b. 30

c. 70

d. 170

| **Utsagn** | Svært uenig | Uenig | Litt uenig | Litt enig | Enig | Svært enig |
| --- | --- | --- | --- | --- | --- | --- |
| 8. Jeg stolte på videoen | 1 | 2 | 3 | 4 | 5 | 6 |
| 9. Jeg følte jeg kunne stole på informasjonen i videoen | 1 | 2 | 3 | 4 | 5 | 6 |

| **Utsagn** | Svært uenig | Uenig | Litt uenig | Litt enig | Enig | Svært enig |
| --- | --- | --- | --- | --- | --- | --- |
| 10. Vitenskap gir oss en bedre forståelse av universet enn religion | 1 | 2 | 3 | 4 | 5 | 6 |
| 11. "I en demon-hjemsøkt verden er vitenskap et lys i mørket." (Carl Sagan) | 1 | 2 | 3 | 4 | 5 | 6 |
| 12. Vi kan bare rasjonelt tro på det som er vitenskapelig bevisbart | 1 | 2 | 3 | 4 | 5 | 6 |
| 13. Vitenskapen forteller oss alt som er å vite om hva virkeligheten består av | 1 | 2 | 3 | 4 | 5 | 6 |
| 14. Alle oppgavene mennesker står overfor kan løses av vitenskapen | 1 | 2 | 3 | 4 | 5 | 6 |
| 15. Den vitenskapelige metoden er den eneste pålitelige veien til kunnskap | 1 | 2 | 3 | 4 | 5 | 6 |
| 16. Den eneste virkelige typen kunnskap vi kan ha er vitenskapelig kunnskap | 1 | 2 | 3 | 4 | 5 | 6 |
| 17. Vitenskap er den mest verdifulle delen av menneskelig kultur | 1 | 2 | 3 | 4 | 5 | 6 |
| 18. Vitenskap er det mest effektive middel for å oppnå sannhet | 1 | 2 | 3 | 4 | 5 | 6 |
| 19. Forskere og vitenskap bør gis mer respekt i det moderne samfunnet | 1 | 2 | 3 | 4 | 5 | 6 |

| **Utsagn** | Definitivt nei | Stort sett nei | Litt nei | Litt ja | Stort sett ja | Definitivt ja |
| --- | --- | --- | --- | --- | --- | --- |
| 20. Har du fulgt koronaanbefalingene? | 1 | 2 | 3 | 4 | 5 | 6 |

| **Utsagn** | Definitivt nei | Stort sett nei | Litt nei | Litt ja | Stort sett ja | Definitivt ja |
| --- | --- | --- | --- | --- | --- | --- |
| 21. Kommer du til å følge anbefalingene? | 1 | 2 | 3 | 4 | 5 | 6 |

22. Hvor gammel er du?

23. Kjønn:

a. Kvinne

b. Mann

c. Jeg foretrekker ikke å svare

24. Bor du i en by:

a. Ja

b. Nei

25. Utdanning:

a. Grunnskole (inntil 10 år)

b. Videregående / fagutdanning (minimum 3 år)

c. Høgskole / Universitet (mindre enn 4 år)

d. Høgskole / Universitet (4 år eller mer)

26. Din inntekt er:

a. Mindre enn 300.000 NOK

b. Mellom 300.000 og 500.000 NOK

c. Mer enn 500.000 NOK

d. Jeg foretrekker ikke å svare

27. Hvis du vil hjelpe oss å finne ut mer og er villig til å delta i fremtidige spørreundersøkelser ber vi deg om å registrere deg ved å fylle ut e-postadressen din.

Questionnaire (English translation)

*Dear participant, thank you for your contribution to this research. We appreciate you taking ten minutes of your time to help us. Watch the short video below. Then a few short questions will follow. Your answers are anonymous and will be analyzed in aggregate form. You will be updated on the findings as soon as the results will be available.*

1. The video you have seen was:

a. artistic

b. scientific

2. The speaker was:

a. a woman

b. a man

| **Statement** | Strongly disagree | Disagree | Slightly disagree | Slightly agree | Agree | Strongly agree |
| --- | --- | --- | --- | --- | --- | --- |
| 3. I understood the content of the video | 1 | 2 | 3 | 4 | 5 | 6 |
| 4. The message was clear | 1 | 2 | 3 | 4 | 5 | 6 |
| 5. I am able to recall the information | 1 | 2 | 3 | 4 | 5 | 6 |
| 6. I am able to tell/spread the message | 1 | 2 | 3 | 4 | 5 | 6 |

7. If R=0.7, how many people will be infected by 100 positive?

a. 7

b. 30

c. 70

d. 170

| **Statement** | Strongly disagree | Disagree | Slightly disagree | Slightly agree | Agree | Strongly agree |
| --- | --- | --- | --- | --- | --- | --- |
| 8. I trusted the video | 1 | 2 | 3 | 4 | 5 | 6 |
| 9. I felt I could trust the information in the video | 1 | 2 | 3 | 4 | 5 | 6 |

| **Statement** | Strongly disagree | Disagree | Slightly disagree | Slightly agree | Agree | Strongly agree |
| --- | --- | --- | --- | --- | --- | --- |
| 10. Science provides us with a better understanding of the universe than does religion. | 1 | 2 | 3 | 4 | 5 | 6 |
| 11. “In a demon-haunted world, science is a candle in the dark.” (Carl Sagan) | 1 | 2 | 3 | 4 | 5 | 6 |
| 12. We can only rationally believe in what is scientifically provable. | 1 | 2 | 3 | 4 | 5 | 6 |
| 13. Science tells us everything there is to know about what reality consists of. | 1 | 2 | 3 | 4 | 5 | 6 |
| 14. All the tasks human beings face are soluble by science. | 1 | 2 | 3 | 4 | 5 | 6 |
| 15. The scientific method is the only reliable path to knowledge. | 1 | 2 | 3 | 4 | 5 | 6 |
| 16. The only real kind of knowledge we can have is scientific knowledge. | 1 | 2 | 3 | 4 | 5 | 6 |
| 17. Science is the most valuable part of human culture. | 1 | 2 | 3 | 4 | 5 | 6 |
| 18. Science is the most efficient means of attaining truth. | 1 | 2 | 3 | 4 | 5 | 6 |
| 19. Scientists and science should be given more respect in modern society. | 1 | 2 | 3 | 4 | 5 | 6 |

| **Statement** | Definitely not | Mostly no | Slightly no | Slightly yes | Mostly yes | Definitely yes |
| --- | --- | --- | --- | --- | --- | --- |
| 20. Have you followed the COVID-19 recommendations? | 1 | 2 | 3 | 4 | 5 | 6 |

| **Statement** | Definitely not | Mostly no | Slightly no | Slightly yes | Mostly yes | Definitely yes |
| --- | --- | --- | --- | --- | --- | --- |
| 21. Are you going to follow the pandemic recommendations? | 1 | 2 | 3 | 4 | 5 | 6 |

22. How old are you?

23. Gender:

a. Woman

b. Man

c. I prefer not to answer

24. Do you live in a city?

a. Yes

b. No

25. Education:

a. Primary school (up to 10 years)

b. High school / vocational training (minimum 3 years)

c. College / University (less than 4 years)

d. College / University (4 years or more)

26. Your income is:

a. Less than 300.000 NOK

b. Between 300.000 and 500.000 NOK

c. More than 500.000 NOK

d. I prefer not to answer

27. If you would like to help us find out more and are willing to participate in future surveys, please register by filling out your email address.
